# The Clinical Society

**Published:** 1896-10-17

**Authors:** 


### The Clinical Society.

Recovery after a penetrating wound of the heart is sufficiently uncommon to give great interest to a case which was related by Mr. W. G. Spencer at the last meeting of the Clinical Society.

The wound, which was inflicted during a struggle, was made by a penknife, which entered at the second left intercostal space. There was severe primary hæmorrhage, and secondary hæmorrhage took place on three occasions, the last being on the seventeenth day. They were sudden and profuse, but apparently fairly easily controlled by plugging. The wound healed six weeks after its infliction, but sixty-nine days after the injury the patient died in consequence of other disorders with which he was affected at the time of the injury, there being found after death chronic tuberculosis of the lungs, cirrhotic and syphilitic disease of the liver, and surgical kidney resulting from old stricture. *Post mortem* examination also showed that the wound had penetrated through the pericardium, and that it extended into the cavity of the right ventricle by a minute puncture. The great interest of the case lies in the fact that notwithstanding the repeated hæmorrhage, showing that the internal wound remained patent for a considerable period, the patient recovered and would have got well permanently had he had reasonably good health. The blood evidently escaped from the right ventricle under a low blood pressure into the pericardium, from which the outburst into the wound took place. A sub-committee, specially appointed for the purpose, accepted Mr. Spencer's view that the wound had penetrated into the ventricle.

A very interesting case of hydatid cyst, and from its size a very uncommon one, was described by Mr. H. B. Robinson at the last meeting of the Clinical Society. The cyst had originated in the liver, and it had extended down into the pelvis and up into the chest. When it was opened by abdominal section the hand introduced into its cavity could be passed upwards through an opening in the diaphragm into the right pleura and into the root of the neck behind the cavicle, the whole forearm and elbow being inserted in so doing. The tumour almost filled the abdomen, the viscera being squeezed into the smallest possible space on the left side, while the whole of the right side of the chest was occupied by it, the lung being completely compressed. The cavity was drained, and the patient recovered.

In commenting on this case, Mr. W. G. Spencer related one in which, on resecting the ribs for the relief of what had appeared to be an empyæma, a large hydatid cyst was met with. By injecting water between it and the pseudo-cyst, the hydatid was removed entire. On further examination a second tumour was discovered in the abdomen which, on laparotomy being performed, was found to lie behind the mesentery. This was treated in the same way, the hydatid removed, and the pseudo-cyst sown up. A third cyst was found one inch within the substance of the right lobe of the liver. This could only be reached by traversing the pleura through the lower ribs. The liver was fixed to the wound and the cyst cavity drained. The patient had recovered, and no further cysts had as yet shown themselves.

